# Association between the SERPING1 Gene and Age-Related Macular Degeneration and Polypoidal Choroidal Vasculopathy in Japanese

**DOI:** 10.1371/journal.pone.0019108

**Published:** 2011-04-19

**Authors:** Isao Nakata, Kenji Yamashiro, Ryo Yamada, Norimoto Gotoh, Hideo Nakanishi, Hisako Hayashi, Akitaka Tsujikawa, Atsushi Otani, Masaaki Saito, Tomohiro Iida, Akio Oishi, Keitaro Matsuo, Kazuo Tajima, Fumihiko Matsuda, Nagahisa Yoshimura

**Affiliations:** 1 Department of Ophthalmology and Visual Sciences, Kyoto University Graduate School of Medicine, Kyoto, Japan; 2 Center for Genomic Medicine/Inserm U.852, Kyoto University Graduate School of Medicine, Kyoto, Japan; 3 Department of Ophthalmology, Fukushima Medical University, Fukushima, Japan; 4 Department of Ophthalmology, Kobe City Medical Center General Hospital, Kobe, Japan; 5 Division of Epidemiology and Prevention, Aichi Cancer Center Research Institute, Nagoya, Japan; French National Centre for Scientific Research, France

## Abstract

**Purpose:**

Recently, a complement component 1 inhibitor (*SERPING1*) gene polymorphism was identified as a novel risk factor for age-related macular degeneration (AMD) in Caucasians. We aimed to investigate whether variations in *SERPING1* are associated with typical AMD or with polypoidal choroidal vasculopathy (PCV) in a Japanese population.

**Methods:**

We performed a case-control study in a group of Japanese patients with typical AMD (n = 401) or PCV (n = 510) and in 2 independent control groups—336 cataract patients without age-related maculopathy and 1,194 healthy Japanese individuals. Differences in the observed genotypic distribution between the case and control groups were tested using chi-square test for trend. Age and gender were adjusted using logistic regression analysis.

**Results:**

We targeted rs2511989 as the haplotype-tagging single nucleotide polymorphism (SNP) for the *SERPING1* gene, which was reported to be associated with the risk of AMD in Caucasians. Although we compared the genotypic distributions of rs2511989 in typical AMD and PCV patients against 2 independent control groups (cataract patients and healthy Japanese individuals), *SERPING1* rs2511989 was not significantly associated with typical AMD (P = 0.932 and 0.513, respectively) or PCV (P = 0.505 and 0.141, respectively). After correction for age and gender differences based on a logistic regression model, the difference in genotypic distributions remained insignificant (P>0.05). Our sample size had a statistical power of more than 90% to detect an association of a risk allele with an odds ratio reported in the original studies for rs2511989 for developing AMD.

**Conclusions:**

In the present study, we could not replicate the reported association between *SERPING1* and either neovascular AMD or PCV in a Japanese population; thus, the results suggest that *SERPING1* does not play a significant role in the risk of developing AMD or PCV in Japanese.

## Introduction

Age-related macular degeneration (AMD) is the leading cause of visual loss in the developed world [1]. Several genes have been reported to be associated with this disease, including complement factor H [2]–[4] and the age-related maculopathy susceptibility 2/HtrA serine peptidase 1 (ARMS2/HTRA1) region [5], [6], and subsequent studies have replicated the association between susceptibility genes and the development of AMD using a different ethnic cohort [7]–[10].

Inner choroidal vascular networks that terminate in polypoidal lesions are defined as polypoidal choroidal vasculopathy (PCV), and are typically visualized by indocyanine green angiography [11]. Whether PCV represents a subtype of neovascular AMD remains controversial; moreover, whether this condition represents inner choroidal vascular abnormalities or is a variety of choroidal neovascularization remains unknown [12]. Previous studies identified several genes that contribute to the development of PCV; however, almost all reported genetic risk factors for PCV are the same as for AMD [13]–[15], and this suggests that AMD and PCV share, at least in part, the same genetic background.

Studies in cohorts from both the United Kingdom and the United States have shown that the complement component 1 inhibitor (*SERPING1*) gene is positively associated with AMD [16]. However, another study in a larger cohort (n = 7723 and 2327) which involved the same population could not replicate the finding of the previous study [17], [18]. Recently, Lee et al. have shown that *SERPING1* is positively associated with AMD in Caucasians [19], but whether this gene is truly associated with AMD remains controversial.

Furthermore, the association of *SERPING1* with AMD has been evaluated also in Asians. Lu et al. examined the association in 194 AMD patients and 285 controls and reported that *SERPING1* is not associated with AMD in the Chinese population [20]. The association between PCV and *SERPING1* has also been evaluated in a smaller Chinese cohort (118 patients and 115 controls), also with negative findings [21]. So far, all Asian studies for *SERPING1* did use smaller cohorts than those of original studies and not consider their statistical power. For evaluating the true gene-disease association, it would be helpful to replicate the positive association reported in previous studies using a different ethnic cohort. The aim of this study, which involved a relatively large number of participants, was to investigate whether the *SERPING1* gene variants are associated with typical AMD or PCV in a Japanese population.

## Materials and Methods

All procedures in this study adhered to the tenets of the Declaration of Helsinki. This study was approved by the Ethics Committee of each institute involved (Kyoto University Graduate School and Faculty of Medicine, Ethics Committee, the Ethical Committee of Fukushima Medical University, the Ethical Committee of Kobe City Medical Center General Hospital, the Ethical Committee of Ozaki Eye Hospital, the Ethical Committee of the Otsu Red Cross Hospital, the Ethical Committee of Nagahama City Hospital, and the Ethical Committee at Aichi Cancer Center). All of the patients were fully informed about the purpose and procedures of this study, and written consent was obtained from each.

In this study, 401 patients with typical AMD and 510 patients with PCV were recruited from the Department of Ophthalmology at Kyoto University Hospital, Fukushima Medical University Hospital, and Kobe City Medical Center General Hospital. The control group included 2 populations: (1) 336 individuals who underwent cataract surgery and had no age-related maculopathy (ARM) (Control 1) were recruited from the Department of Ophthalmology, Kyoto University Hospital, Ozaki Eye Hospital, Japanese Red Cross Otsu Hospital, and Nagahama City Hospital; and (2) 1194 healthy individuals who were recruited from the Aichi Cancer Center Research Institute as the general population control (Control 2). AMD and ARM were defined according to the International Classification System for ARM and AMD [22]. The diagnosis of PCV was based on indocyanine green angiography, which showed a branching vascular network that terminated in polypoidal swelling. Typical AMD were late AMD which showed classic choroidal neovascularization (CNV), occult CNV, or both. All diagnoses were made by 3 retina specialists (K.Y., A.T., and A.O.); a fourth specialist (N.Y.) was consulted when the subtype classification could not be decided on by the initial 3 reviewers. All of the subjects were unrelated and were of the Japanese descent.

Genomic DNAs were isolated from the peripheral blood of the subjects by using a DNA extraction kit (QuickGene-610L, Fujifilm, Minato, Tokyo, Japan). The samples of all the patients with typical AMD and PCV and of cataract patients were genotyped using a Taqman single nucleotide polymorphism (SNP) assay with the ABI PRISM 7700 system (Applied Biosystems, Foster City, CA). The individuals recruited from the Aichi Cancer Center Research Institute were genotyped using Illumina Human-Hap 610 chips (Illumina Inc., San Diego, CA).

Linkage disequilibrium (LD) structures across the *SERPING1* gene were compared between the Caucasian and Japanese populations, using genotype data retrieved from the HapMap CEU and JPT data sets [23]. The retrieved data were loaded into Haploview to estimate LD parameters and to identify haplotype blocks [24]. Deviations in genotype distributions from the Hardy–Weinberg equilibrium (HWE) were assessed using the HWE exact test. Statistical analyses for differences in the observed genotypic distribution were performed by the chi square test for trend; logistic regression analysis was performed for age and gender adjustments. The statistical power calculation was performed using QUANTO version 1.2 [25]. P values less than 0.05 were considered statistically significant.

## Results

**Figure 1:**
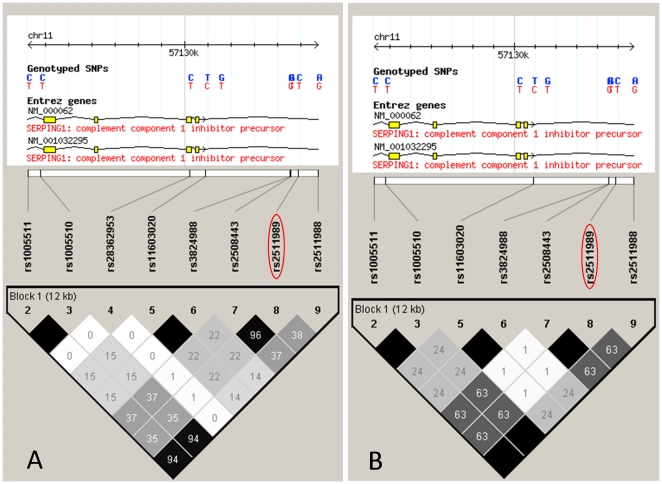
Linkage disequilibrium (LD) structure across the complement component 1 inhibitor (*SERPING1*) gene in Caucasian and Japanese populations. Genotype data were retrieved from HapMap CEU (Utah residents with ancestry from northern and western Europe; A) and JPT (Japanese in Tokyo, Japan; B) data sets. Haplotype blocks were determined using the “four-gamete rule” option in Haploview; all HapMap single nucleotide polymorphisms on *SERPING1* gene are in the same block in both populations. Each box provides estimated statistics of the coefficient of determination (r^2^), with darker shades representing stronger LD.

The demographic details of the study population are presented in Table 1. Because all SNPs of the *SERPING1* gene are in the same haplotype block, rs2511989 was selected as the haplotype-tagging SNP; rs2511989 was reported to be associated with the risk of AMD in previous studies [16], [19] (Fig. 1). Details of allele and genotype counts and summary statistics for rs2511989 are shown in Table 2. The success rate of genotyping of rs2511989 was 98.1%, and the distributions of the genotypes for all study groups were in the Hardy–Weinberg equilibrium (P>0.05). Although we compared the genotype distributions of rs2511989 in typical AMD and PCV patients against 2 independent control groups (cataract patients without ARM and healthy Japanese individuals), *SERPING1* rs2511989 was not significantly associated with typical AMD (P = 0.932 and 0.513, respectively); furthermore, it was not significantly associated with PCV (P = 0.505 and 0.141, respectively). After correction for age and gender differences based on a logistic regression model, the difference in the genotype distributions remained insignificant (P>0.05). Table 3 shows the odds ratios adjusted for age and gender under various genetic models. We could not find a significant association in any genetic model.

**Table 1:** Characteristics of the Study Population.

		Cases		Controls	
		tAMD	PCV	Control 1*	Control 2†
No. of participants		401	510	336	1194
Age	Mean ± SD	77.38±8.39	74.98±7.77	74.16±8.42	50.34±15.9
Gender	Men	287	372	142	493
	Women	114	138	194	701

tAMD, typical age-related macular degeneration; PCV, polypoidal choroidal vasculopathy; SD, standard deviation.

*Cataract patients without age-related maculopathy.

†Healthy Japanese individuals.

**Table 2:** *SERPING1* rs2511989 Genotypic Distributions and Results of Association Tests and Power Analysis.

						vs Control 1			vs Control 2		
		GG	GA	AA	MAF	P Value	Adjusted P*	Power†	P Value	Adjusted P*	Power†
Cases	tAMD	293	102	6	0.142	0.932	0.687	93.6%	0.513	0.860	99.3%
	PCV	380	125	5	0.132	0.505	0.855	95.7%	0.141	0.678	99.2%
Controls	Control 1	248	76	10	0.144	-	-	-	-	-	-
	Control 2	859	308	27	0.152	-	-	-	-	-	-

tAMD, typical age-related macular degeneration; PCV, polypoidal choroidal vasculopathy; MAF, minor allele frequency.

*Adjusted for age and gender.

†Statistical power for detecting the association reported in the previous study (odds ratio 0.63).

**Table 3:** Odds Ratios in Various Genetic Models.

		Adjusted Odds Ratio (95% Confidence Interval)*	
Group	Model	vs tAMD	vs PCV
Control 1	Additive	0.938 (0.687–1.281)	0.972 (0.72–1.312)
	Dominant	1.283 (0.746–2.204)	0.598 (0.338–1.056)
	Recessive	0.934 (0.783–1.114)	1.283 (0.746–2.204)
Control 2	Additive	1.034 (0.716–1.491)	0.933 (0.673–1.294)
	Dominant	0.940 (0.470–1.879)	0.709 (0.349–1.440)
	Recessive	1.025 (0.839–1.254)	0.983 (0.823–1.174)

*Adjusted for age and gender.

tAMD, typical age-related macular degeneration; PCV, polypoidal choroidal vasculopathy.

Next, we calculated our statistical power to detect an association of a risk allele with the odds ratio reported in the previous study that investigated the association of rs2511989 with developing AMD. When we targeted the original study reported by Ennis (odds ratio 0.63) [16], our sample size had more than 90% power to detect the association (Table 2). In addition, the statistical power calculation revealed that our sample size could detect the gene-disease association for an odds ratio of 0.797 by more than 80%.

## Discussion

In the present study, we investigated whether *SERPING1* gene variants are associated with typical AMD or with PCV in a Japanese population. We selected rs2511989 as the haplotype-tagging SNP, because this has been reported to be positively associated with the risk of AMD in Caucasians. The results of this study showed that *SERPING1* rs2511989 was not associated with the risk for typical AMD in a Japanese population; thus, the results did not support the hypothesis that an association between the *SERPING1* gene and AMD exists. Our sample size had more than 90% power to detect the association determined in the previous study in a Caucasian cohort (odds ratio 0.63) [16]. Furthermore, we found no evidence to support the role played by *SERPING1* rs2511989 in the susceptibility to PCV, and this finding is in agreement with that of the previous study in a Chinese population [21].

The reported association between AMD and *SERPING1* rs2511989 is shown in Table 4. The size of our Japanese cohort was similar to that of the original study [16]. Furthermore, the statistical power calculation revealed that our sample size could detect the gene-disease association for an odds ratio of 0.797 by more than 80%. Had there been a true protective effect of *SERPING1* gene variants for developing AMD at the same level as was reported in previous studies [16], [19], the statistical power of our study would have detected such an association. Differences in the ethnicities of subjects might be 1 reason for the difference observed between the results of this study in a Japanese cohort and those of the previous study in a Caucasian cohort. Frequency of the minor allele of rs2511989 was reportedly greater in the earlier study in a Caucasian population than that of the present study in a Japanese population. In fact, in reference to the allele frequency data from the HapMap, all genetic variants across the *SERPING1* gene showed smaller minor allele frequency in Japanese than in Caucasians.

**Table 4:** Comparison of Association Observed between AMD and *SERPING1* rs2511989.

Subject Group		Current Study (JP)			Mayo Subjects (US)		AREDS Subjects (US)		Ennis et al. (UK)		Ennis et al. (US)		Lee et al. (US)		Lu et al. (CH)	
Subjects		Case	Control 1	Control 2	Case	Control	Case	Control	Case	Control	Case	Control	Case	Control	Case	Control
No. of participants		401	336	1194	470	310	1221	295	479	479	248	252	556	256	194	285
Allele count	G	688	572	2026	569	363	1435	357	597	500	322	282	669	283	336	493
	A	114	96	362	371	257	1007	233	355	454	174	222	413	229	52	69
Genotype count	GG	293	248	859	179	103	436	115	191	132	100	79	213	74	147	215
	GA	102	76	308	211	157	563	127	215	236	122	124	273	135	42	63
	AA	6	10	27	80	50	222	53	70	109	26	49	70	47	5	3
MAF		0.142	0.144	0.152	0.395	0.415	0.412	0.395	0.373	0.475	0.351	0.441	0.382	0.447	0.134	0.123
P values		-	0.932	0.513	-	0.46	-	0.41	-	5.4×10^−6^	-	0.0037	-	0.011	-	0.61

MAF, minor allele frequency.

Another possible explanation for the differences between our findings and those of other studies in different ethnic cohorts may include a difference in the phenotypes of AMD. Numerous studies have reported that distinguishing features of Asian AMD include male predominance, unilateral presentation, comparatively low incidence of soft drusen, and greater prevalence of neovascular AMD and PCV [26]–[29]. To address these concerns, we classified AMD patients into those with typical AMD and those with PCV, but the possible hidden differences in the phenotypes cannot be excluded. Alternatively, considering the fact that genetic variants that are associated with a particular disease in 1 population may not necessarily be associated in another population [30]–[32]; moreover, it is possible that gene-disease association of *SERPING1* in populations from East Asia is very weak or absent as compared with Caucasian populations.

In this study, we used general population-based controls (Control 2). The possibility exists that some of the eyes in the control 2 group might have or develop AMD or PCV, and this might be a possible explanation for the negative results in this study. However, because the prevalence of AMD in the general population is estimated to be 0.5% in the Japanese population [33], the loss of the statistical power of association analysis must be negligible. In addition, we also performed a subset analysis on controls 2 with 55 years of age or older to minimize the possibility that some of the eyes in the control group might develop AMD or PCV. However, no new significant differences in the genotypic distributions were found in the current study (data not shown). Thus, we concluded that the result of the analysis using control 2 is valuable as reference data which supports a lack of association between *SERPING1* and both typical AMD and PCV in a Japanese population. Another limitation is about geographical difference of Control 1, which may influence genetic background of the participants. However, because the Japanese population has been reported to have a rather small genetic diversity, according to data from the SNP discovery project in Japan [34], geographical difference should not be affect our statistical results.

In conclusion, this study showed a lack of association between *SERPING1* and both typical AMD and PCV in a Japanese population; thus, the results suggest that *SERPING1* does not play a significant role in the risk of developing AMD or PCV in Japanese.
